# Biophysical Diffusion MRI Models Better Identify White Matter Tracts in Edema

**DOI:** 10.3390/tomography12060078

**Published:** 2026-05-25

**Authors:** Isaac E. Prentiss, Sasha Hakhu, Jennapher Lingo VanGilder, Parvathy Hareesh, Andrew Hooyman, Jason Yalim, Justin Hines, Gabe LaFond, Edward Ofori, Leslie C. Baxter, Yuxiang Zhou, Leland S. Hu, Kurt G. Schilling, Scott C. Beeman

**Affiliations:** 1 School of Biological and Health Systems Engineering, Arizona State University, Tempe, AZ 85287, USAhooyman@chapman.edu (A.H.); 2 Research Computing, Arizona State University, Tempe, AZ 85287, USA; 3 Department of Radiology, Mayo Clinic, Phoenix, AZ 85054, USA; 4 College of Health Solutions, Arizona State University, Tempe, AZ 85287, USA; 5 Department of Psychiatry and Psychology, Mayo Clinic, Phoenix, AZ 85054, USA; 6 Department of Radiology, Vanderbilt University Medical Center, Nashville, TN 37232, USA; 7 John Shufeldt School of Medicine and Medical Engineering, Arizona State University, Tempe, AZ 85287, USA

**Keywords:** NODDI, SM, DTI, edema, diffusion MRI, white matter, tractography

## Abstract

Background/Objectives: White matter (WM) tract detection is critical in the presurgical planning of tumor resection. However, standard-of-care imaging techniques including T_1_-weighted, T_2_-weighted, and Diffusion Tensor Imaging (DTI) often fail to identify WM tracts within edematous regions. In T_1_/T_2_-weighted imaging, edema increases extracellular water and reduces tissue contrast, and in diffusion-weighted imaging, edema elevates isotropic diffusion, reducing sensitivity to anisotropic diffusion along WM tracts. Advanced biophysical diffusion modeling techniques such as Neurite Orientation Dispersion and Density Imaging (NODDI) and the Standard Model (SM) address this limitation by compartmentalizing the diffusion signal into free-water, intra-neurite, and extra-neurite contributions. Here, we test if biophysical multi-compartment models can robustly identify WM tracts and recover tractography streamlines within edematous regions. Methods: In this study, we use multi-shell diffusion-weighted MRI data obtained from patients with meningiomas—a pathology allowing for isolation of the effects of edema without the confounding effects of tumor cell invasion. We compared FA from standard and free-water-corrected DTI, the orientation dispersion index (ODI) from NODDI, and P_2_ (a scalar descriptor of fiber orientation coherence) from the SM fODF in edematous and unaffected contralateral WM regions. As a proof of concept, we visually evaluated the tractography performance across models. Results: Our results show that (1 − ODI) and P_2_ values in edema remained close to within-subject contralateral measurements, contrasting with substantial reductions in FA and FW-FA. (1 − ODI) showed a small but statistically significant increase in edema (~8%, *p* = 0.02), while P_2_ was unchanged. Conclusions: These results highlight the potential of biophysical diffusion models for preoperative mapping in edema.

## Simple Summary

Brain tumors often cause nearby tissue to swell, and this swelling can hide the appearance of the bundles of nerve fibers, called white matter, that carry signals between different regions of the brain on standard MRI brain imaging. This makes it difficult for surgeons to know exactly where these fibers are when planning surgery. In this study, we tested whether more advanced diffusion MRI methods, which account for the way in which water moves around in different microscopic parts of the brain, could better identify white matter through the swelling. In five patients with benign meningioma tumors, the advanced methods better traced white matter pathways than standard methods. These findings suggest that newer diffusion MRI approaches may improve preoperative planning for brain surgery in regions affected by swelling.

## 1. Introduction

Precise identification of white matter (WM) tracts is critical in the planning of brain surgery, as any damage to the WM can lead to irreversible deficits, including motor, language, or cognitive impairments [1,2]. Magnetic resonance imaging (MRI) techniques, such as T_1_-weighted (T_1_) and T_2_-weighted (T_2_) imaging, have traditionally formed the foundation for the mapping of such structures; however, these image volumes often struggle to delineate WM in cases of vasogenic edema due to a lack of relaxation-driven contrast in these regions [3,4].

Diffusion MRI (dMRI) has long been used to detect and track WM in the brain [5,6,7,8,9,10,11] and the peripheral nervous system [12,13,14,15]. dMRI is sensitive to the displacement of water molecules within the constraints of the complex tissue microenvironment and can thus be used to identify and make quantitative inferences of the tissue microstructure (e.g., WM tract directionality, axon and dendrite density, and orientation complexity). Diffusion Tensor Imaging (DTI), the most ubiquitous quantitative dMRI method, fits a tensor to a series of diffusion-weighted image (DWI) volumes and calculates eigenvectors and eigenvalues. From these, the fractional anisotropy (FA) scalar metrics are calculated, which quantify the degree of anisotropy of proton displacement from 0 (perfectly isotropic) to 1 (perfectly anisotropic) [16]. The diffusion-driven displacement of water is typically anisotropic in white matter (WM) due to the coherent orientation of myelinated axon bundles and restriction of water displacement within that geometry, characterized by a higher FA value compared to gray matter (GM) and cerebrospinal fluid (CSF). DTI forms the foundation of most diffusion MRI-based studies of WM in the brain and presurgical mapping. This measure of anisotropy can then be utilized to make diffusion MR tractography calculations based on the primary eigenvectors, which track the location and trajectories of WM fibers to assist neurosurgeons in preoperative planning [17].

Notably, the DTI calculation struggles to identify WM in cases of peritumoral edema, as it only fits a single diffusion tensor representing the displacement of water for each voxel and thus conflates the anisotropic diffusion within WM tissue with the isotropic diffusion associated with the edema-associated free water [18,19,20,21,22,23]. Restated, the increased interstitial water volume associated with peritumoral edema results in the averaging of diffusion signal properties of tissue and free water, driving FA measurements down below those characteristic of WM [24,25]. This is a known consequence of the DTI calculation and its associated single-shelled dMRI acquisition scheme, which allows for only one diffusivity calculation from two data points (typically b = 0 and b = 1000 s/mm^2^). More sophisticated implementations of DTI have been explored to address this limitation, such as free-water-corrected DTI (FW-DTI) [24], which fits a bi-tensor model to account for both tissue as well as freely diffusing water compartments to calculate the FA corrected for free water contamination (FW-FA). FW-DTI has been demonstrated as a promising alternative to DTI metrics in detecting WM in edematous environments, but it is an ill-posed problem without either spatial regularization or multiple shells, and challenges persist in adequately removing free-water partial volume effects [24,26].

To better quantify the brain tissue microstructure from dMRI data, multi-shell biophysical models have been developed, including (but not limited to) Neurite Orientation Dispersion and Density Imaging (NODDI) [27] and the Standard Model (SM) [28]. These approaches provide compartment-specific characterization of the tissue microstructure by separating the diffusion signal into free water, intra-neurite water, and extra-neurite water. NODDI models the free-water compartment as a sphere, intra-neurite water as zero-radius “sticks” (represented by a Watson distribution), and extra-neurite water (represented by an anisotropic tensor), each with fixed diffusivity values. Output parameter estimates of NODDI include orientation vectors that specify the principal direction of anisotropy within a voxel (shared by the tensor and the Watson distribution) and descriptive scalar values including the Neurite Density Index (NDI), Orientation Dispersion Index (ODI), and isotropic volume fraction (fISO). This study places significant emphasis on the ODI scalar value, which is derived from the Watson distribution and scaled from 0 to 1. Low ODI values indicate tightly aligned fibers (high coherence), while high values indicate broadly dispersed fibers (low coherence). In WM tracts, ODI is therefore expected to remain comparatively robust to edema, because the joint compartmental fit better isolates the stick distribution from isotropic and extra-neurite signals.

The SM framework adopts a more general biophysical formulation of a similar form to NODDI. It also models the free-water compartment as a sphere, intra-neurite water as zero-radius “sticks” (the angular distribution of which is represented in the general spherical harmonic (SH) basis instead of a Watson distribution), and extra-neurite water (represented by an anisotropic tensor). The SM builds on models like NODDI and makes fewer assumptions about compartment diffusivities [28]. The SM’s primary outputs include intra-axonal and extra-axonal signal fractions, intra-axonal axial diffusivity, extra-axonal axial and radial diffusivity, and a fiber orientation distribution function (fODF), which carries orientation information in the SH basis that is compatible with probabilistic tractography algorithms. This study primarily focuses on the second-order rotational invariant of the fODF (P_2_), which serves as a scalar descriptor of fiber orientation dispersion [28].

In both the NODDI and SM cases, it should be noted that these signal compartments do not necessarily map cleanly onto distinct biological compartments despite the naming conventions. The “free-water” component typically captures ventricular water and larger extracellular parenchymal spaces, while the “extra-neurite/extra-axonal” component captures parenchymal extracellular water and soma water. The “intra-neurite/intra-axonal” component is the fraction of the signal with diffusion properties expected of zero-radius cylinder (or stick) geometries generally thought to reflect myelinated axons.

Here, we aim to evaluate the DTI, FW-DTI, NODDI, and SM performance in detecting WM tracts within peritumoral edema in subjects bearing non-infiltrative meningioma tumors, a pathology which lacks tumor invasion into WM tracts and thus isolates the effects of edema [29,30,31,32,33,34]. We hypothesize that ODI (here, reflected as (1 − ODI) to retain consistency with the directional interpretation of all metrics) and P_2_ will remain more robust to free-water contamination and that FW-corrected FA will recover anisotropy relative to standard FA, with each providing improved identification of WM tracts through regions of edema. Similarly, we hypothesize that the primary reconstructed orientations derived from FW-DTI (eigenvectors), NODDI (Watson mean direction or, equivalently, the principal eigenvector of NODDI’s extra-neurite tensor), and the SM (fODF peaks) will likewise be less affected by edema than DTI’s principal eigenvector. As a proof-of-principle analysis, we test whether tractography generated with the scalar and directional outputs of FW-DTI, NODDI, and the SM will maintain greater continuity through edematous regions than tractography based on FA. By disentangling edema-related isotropic water signals from neurite-specific features in a non-infiltrative tumor model, this study seeks to identify the diffusion framework that most reliably detects WM and supports tractography near surgical targets. Such methods will better guide preoperative planning toward models whose parameters are less confounded by edema and more reliably identify WM tracts in these regions.

## 2. Materials and Methods

### 2.1. Participant Population

**Figure 1:**
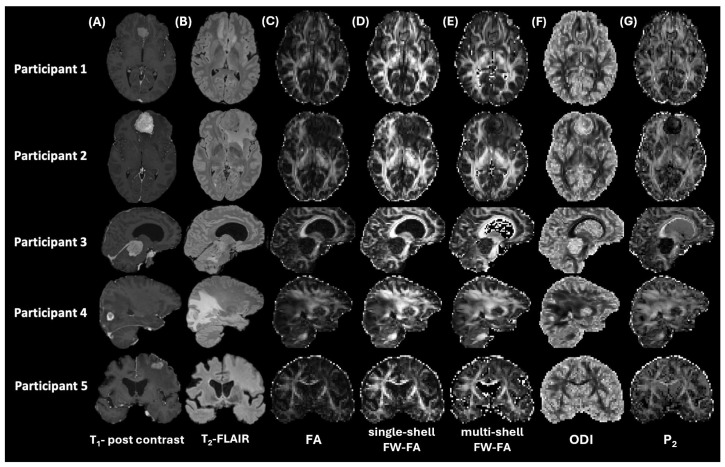
Representative (**A**) post-contrast T_1_-weighted images, (**B**) T2-weighted FLAIR images, (**C**) FA maps, (**D**) single-shell FW-FA maps, (**E**) multi-shell FW-FA maps, (**F**) ODI maps, and (**G**) P_2_ maps are shown. Post-contrast T_1_-weighted images best reflect tumor location, and T_2_-weighted FLAIR images best reflect tumor plus edema location. DTI’s FA (where WM is typically represented by a brighter signal intensity) fails to identify WM tracts through regions of edema (seen as hyperintense signal traced in T_2_-FLAIR images), whereas NODDI’s ODI (where WM is represented by a darker signal intensity) retains WM structure irrespective of edema presence. Similarly, the SM’s P_2_ map (where WM is typically represented by a brighter signal intensity) succeeds. Representative image planes were chosen on a per-patient basis to best reflect the lesion.

Five adults aged 62 to 77 years old bearing radiologically confirmed meningiomas were included in this study. Participant data were acquired and anonymized by the Mayo Clinic, Phoenix, Arizona, and processed and analyzed at Arizona State University. All experimental procedures were approved by each institution’s Institutional Review Board. Tumor locations included: participant 1—frontal lobe; participant 2—frontal lobe; participant 3—left anterior limbic lobe; participant 4—left occipital lobe; and participant 5—left frontal lobe (Figure 1 shows a panel of these participants’ tumor locations (in a post-contrast T_1_), edema regions (in a T_2_-FLAIR), and their associated FA, FW-FA, ODI, and P_2_ maps). All meningioma cases included in this study were histopathologically confirmed primary tumors. These comprised WHO Grade I (transitional and angiomatous subtypes)—subtypes were determined through standard histological evaluation, including immunohistochemical staining (e.g., SSTR2, GFAP), following surgical resection. An independent review was performed by a board-certified neuroradiologist to additionally confirm no radiologic evidence of Wallerian degeneration or encephalomalacia in adjacent WM. Additionally, five healthy adults aged 75 to 88 years old were included to establish tractography threshold values.

### 2.2. Neuroimaging Data Acquisition

Imaging data were acquired on a 3T Siemens (Siemens Healthcare, Erlangen, Germany) scanner using a 20-channel coil. Acquired imaging data include: (i) a three-dimensional (3D) axial T_1_-weighted magnetization-prepared rapid acquisition gradient echo (MPRAGE) sequence, (ii) a 3D post-Gadolinium (Gd) T_1_-weighted MPRAGE sequence (TR/TE 1900/2.02 ms, 208 slices), (iii) a 3D T_2_-weighted fluid-attenuated inversion recovery (FLAIR) sequence (TR/TE 5000/389 ms, 192 slices), and (iv) multi-shell diffusion-weighted acquisitions with the following parameters: b-values (s/mm^2^) = 0 (5 volumes), 200 (6 directions), 300 (6 directions), 500 (6 directions), 750 (12 directions), 1000 (18 directions), 1500 (24 directions), 2000 (30 directions), and 2500 (36 directions). A total of 42 IVIM-sensitive volumes were acquired between b-values of 0 and 200 (TR/TE 3664/95.6 ms, 185 volumes). The total acquisition time for our multi-shell diffusion-weighted imaging (DWI) protocol was 14 min. Note that these data were acquired as part of a larger and separate study of diffusion MRI in brain tumors and not all shells are used (see below). All MR images were screened for neuropathology by a licensed neuroradiologist prior to analysis.

### 2.3. Diffusion Neuroimaging Preprocessing

Data were corrected for noise (MRtrix3, v3.0.8) [35,36], field map distortion (Synb0) [37], and eddy currents (MRtrix3 and FSL v6.0.7.19) [38]. Specifically, for noise correction, we used MRtrix3’s *dwidenoise* tool, which applies a data-driven approach to reduce noise based on a local estimate of the data’s noise characteristics. This tool is based on the MPPCA [36] method. We applied Synb0-DISCO to generate an undistorted b0 image that was used with *topup* to correct for geometric image distortions caused by susceptibility effects. Additionally, *eddy* was applied to correct for eddy current distortions, motion artifacts, and slice-to-volume misalignments, while also performing outlier detection to identify slices affected by subject movement. DTI (using FSL’s *DTIFIT* toolbox) [39], single-shelled FW-DTI (using a Python toolbox implementing a single-shell regularized gradient descent approach) (https://www.python.org) [40], multi-shelled FW-DTI (using a Python toolbox from DIPY) [41], SM (using a MATLAB toolbox developed at New York University) [42] (MATLAB R2026a; The MathWorks, Inc., Natick, MA, USA), and Watson-NODDI (using the NODDI MATLAB toolbox) [43] models were applied to preprocessed data to generate diffusion parameter maps, with FA, single- and multi-shelled FW-FA, ODI, and P_2_ maps representing directionality-specific scalar metrics from each method, respectively. For consistency with the directional interpretation of FA, FW-FA, and P_2_, all of which take higher values in more coherent WM, we report (1 − ODI) rather than ODI in all instances but the ODI maps. This convention also matches how ODI is used as a gating field for FACT-based tractography (Section 2.5).

As part of a secondary analysis examining water volume fraction effects on WM, fISO and NDI maps were generated from NODDI, and free-water signal fraction (fw) and intra-axonal signal fraction (f) maps were generated from the SM. Diffusion volumes with b-values of 0 and 1000 s/mm^2^ were used for DTI and single-shelled FW-DTI. b = 0 and shells with b ≥ 750 s/mm^2^ were used to fit NODDI, the SM, and multi-shell FW-DTI. Volumes with 0 < b < 750 were excluded from this analysis to (i) mitigate the effects of convection/flow in the capillary, which is most severe at b ≤ 300 s/mm^2^ but contributes non-negligibly to b = 500 s/mm^2^—these fast-moving water compartments produce apparent diffusivities greater than that of free water at 37 °C (D = 3 × 10^−3^ mm^2^/s) and are not accommodated by any of the models considered here—and (ii) conform to standard practice in the literature, which nearly universally uses shells with b ≥ 700 s/mm^2^ for NODDI and SM fits [27,28,44]. Python scripts developed in lab and derived heavily from the *select_dwi_vols* [39] tool were used to extract specific b-value shells from the multi-shell dataset for each respective model. All volumes were then co-registered to the pre-contrast T_1_-weighted volume for region of interest (ROI) analysis.

### 2.4. ROI Segmentation and Processing

**Figure 2:**
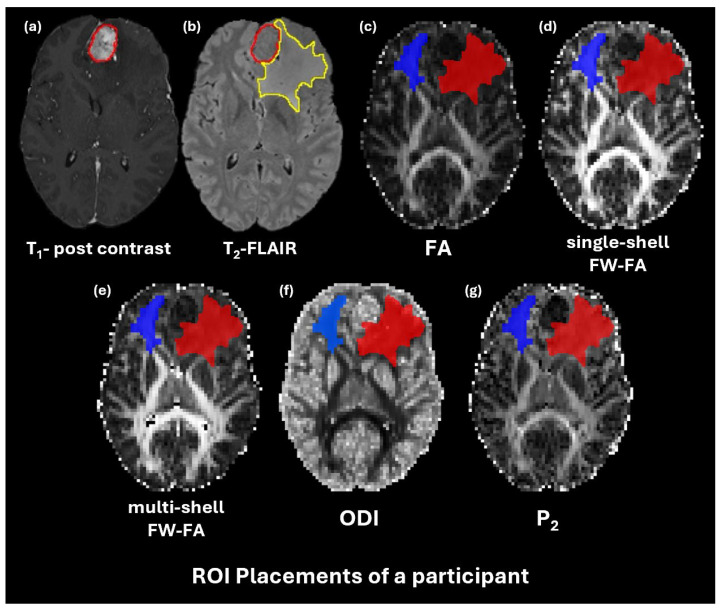
Example ROI placements on T_1_ post-Gd and T_2_ FLAIR images and FA and ODI maps. (**a**) T_1_ post-Gd image with tumor contour (red), (**b**) T_2_ FLAIR image with tumor (red) and edema (yellow) contours, (**c**) FA, (**d**) single-shell FW-FA, (**e**) multi-shell FW-FA, (**f**) ODI maps, and (**g**) P_2_ maps with edema (in red) and the contralateral region (in blue) highlighted. For each participant, the tumor contour was identified and segmented using the T_1_-weighted post-contrast volume and was then overlaid on the T_2_-weighted FLAIR image; then, the peritumoral edematous contour was identified and segmented based on the hyperintense signal in the T_2_ FLAIR. The region highlighting extra-tumoral edema was then derived by subtracting the two segmentations (i.e., FLAIR region of interest minus the post-contrast T_1_-weighted region of interest). As a control, healthy-appearing contralateral white matter tracts were identified by first flipping the edema ROI over the midline and then manually adjusting the outline to include only white matter containing voxels. A T_1_-weighted image was used to verify locations of contralateral WM tract ROIs. These ROIs were then overlaid on the diffusion maps, and the location of each ROI was confirmed by a licensed neuroradiologist. Participants’ images were then co-registered to their T_1_-weighted image.

For each participant, the tumor ROI was manually segmented using the post-contrast T_1_-weighted volume. The total edema ROI (including tumor and peritumoral edema) was identified and segmented based on the hyperintense signal of the T_2_-FLAIR. The extra-tumoral edema ROI was derived by subtracting the tumor segment from the total edema segment. As a control, healthy-appearing contralateral white matter tracts were identified by first reflecting the extra-tumoral edema ROI over the midline and then manually adjusting the outline using both the pre-contrast T_1_-weighted image and the T_2_-FLAIR image as anatomical guides. Specifically, the contralateral ROIs were adjusted on an individual basis to ensure (i) restriction to only WM tracts and (ii) consistency with visible anatomical landmarks (e.g., ventricular boundaries, major WM tracts, etc.), accommodating any tumor-induced mass effect or local deformation. These ROIs were then overlaid on diffusion scalar maps (FA, FW-FA, ODI, fISO, NDI, P_2_, fw, and f), and the location of each ROI was confirmed by a board-certified neuroradiologist (see edema appearance in these image volumes in Figure 2). Voxel values were extracted from edematous and contralateral ROIs using *fslstats*.

### 2.5. Tractography

For the primary tractography comparison, we implemented “Fiber Assigned by Continuous Tracking” (FACT) [45] using MRtrix3’s *tckgen* package [46]. Here, the primary direction vector scaled by directionality-specific scalar metrics (DTI, single- and multi-shelled FW-DTI, NODDI, and the SM) was used as the input image, along with the DWI brain mask as the seed image. For standard and free-water variants of DTI, primary eigenvectors saved as outputs were scaled by their respective FA outputs to be used as tractography inputs. For NODDI-based tractography, fitted primary orientation vectors (from concatenating Cartesian unit vector outputs) were utilized alongside ODI. Additionally, ODI was inverted (1 − ODI) before use as the gating field, since FACT interprets higher input values as indicating greater directional coherence (the directional inverse of how ODI is conventionally interpreted). To ensure a fair comparison for a FACT-based analysis, the SM’s fODFs were vectorized based on the direction of peak fiber probability (*sh2peaks*), converted into unit vectors, and scaled by P_2_.

Initiation and cutoff thresholds were optimized heuristically for deterministic fiber tracking by maximizing the Dice [47] similarity between differing thresholds of anisotropy scalar maps (FA, FW-FA maps, (1 − ODI), and P_2_) for an independent cohort of five healthy age-matched subjects separate from the meningioma subjects. Scalar maps were co-registered to T_1_-weighted images (*flirt*), and segmentation (*fast*) [48] was performed on T_1_ images to extract a computed WM segmentation. Scalar maps were then thresholded in T_1_ space, and the threshold value that maximized the Dice similarity coefficient between the segmented WM and the respective scalar map was used for subsequent fiber tracking. Mean scalar map optimal thresholds were 0.25 for FA; 0.42 for single-shelled FW-FA; 0.38 for multi-shelled FW-FA; 0.66 for (1 − ODI); and 0.31 for P_2_. Ventricle masks were created to exclude any streamlines in the region of ventricles. A total of 10,000 streamlines were generated for all methods used here. Tractography results were independently reviewed by a board-certified neuroradiologist, who confirmed anatomic plausibility of the streamline trajectories.

Secondary to the deterministic tractography comparison, we examined the potential of fODF-based probabilistic fiber tracking approaches using multi-shell, multi-tissue constrained spherical deconvolution (MSMT-CSD) alongside probabilistic tractography driven by the SM’s fODF output. MSMT-CSD fODFs were estimated using the default workflow in MRtrix3, with characteristic response functions estimated using the default algorithm (*dwi2response*) [49], followed by the default multi-shell deconvolution algorithm (*dwi2fod msmt_csd*) [50]. Probabilistic fiber tracking was performed using the “Second Order Integration over Fiber Orientation Distributions” (iFOD2) algorithm [51]. Default input parameters were used, along with the DWI brain mask used as a seed image and 10,000 streamlines generated. The default cutoff thresholds of 0.1 were additionally utilized for CSD, while cutoff thresholds of 0.2 were used for SM fODF tractography. To assess qualitative probabilistic SM results on the cutoff thresholds, SM tractography was repeated across values of 0.05, 0.1, 0.15, 0.2, 0.25, and 0.3. A threshold of 0.2 was heuristically found to qualitatively preserve streamline continuity while mitigating spurious fibers from a cutoff threshold that is too liberal. Results were further confirmed to be anatomically feasible by a board-certified neuroradiologist.

### 2.6. Statistical Testing

Quantitative differences between edematous and contralateral regions were assessed using FA, FW-FA, (1 − ODI), and P_2_ metrics derived from DTI, FW-DTI, NODDI, and the SM, respectively. Percentage differences were computed for each ROI using a standardized formula:

(1) $$\%\;\mathrm{difference}=\frac{{\mathrm{metric}}_{\mathrm{edematouse}}-{\mathrm{metric}}_{\mathrm{non}-\mathrm{edematous}}}{{\mathrm{metric}}_{\mathrm{non}-\mathrm{edematous}}}*100\%$$

Statistical analyses were conducted using R version 4.3.3 [52], where the normality of the scalar metrics’ (FA, FW-FA, (1 − ODI), P_2_, fISO, NDI, fw, and f) distributions was assessed using the Shapiro–Wilk test. Paired t-tests were performed between scalar metric distributions in edematous and non-edematous hemispheres for each metric. A total of nine comparisons combining ROIs for all five participants were performed, and *p*-values less than 0.05 were considered significant.

## 3. Results

### 3.1. Statistical Comparison Details

Quantitative differences between edematous and contralateral brain regions were evaluated using FA, FW-FA, (1 − ODI), and P_2_ metrics derived from DTI, FW-DTI, NODDI and SM approaches. Percentage differences were calculated using Equation (1), and the results are summarized in Table 1.

**Figure 3:**
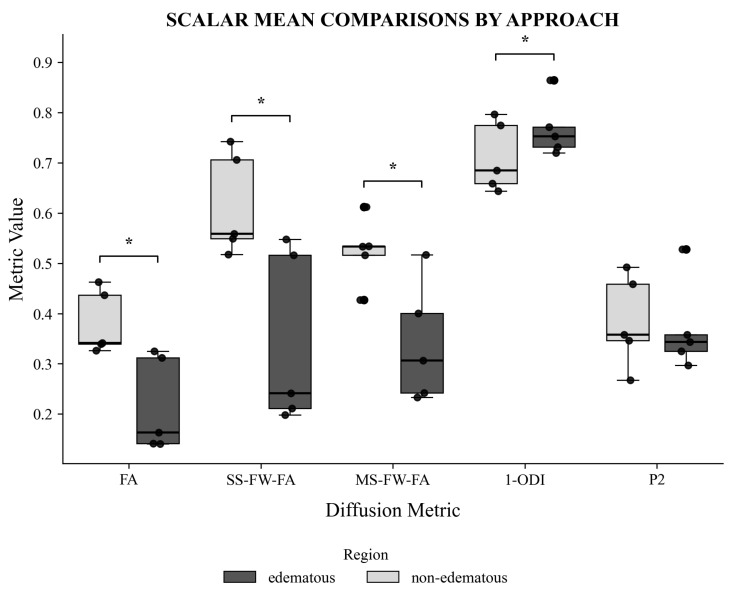
Statistical comparisons between aggregated edematous and non-edematous regions by diffusion scalar metric. * *p* < 0.05.

Paired *t*-tests showed that FA (β = 0.17, 95% CI = [+0.05; +0.29], *p* = 0.018), single-shelled FW-FA (β = 0.27, 95% CI = [+0.06; +0.49], *p* = 0.025), multi-shelled FW-FA (β = 0.18, 95% CI = [+0.08; +0.28], *p* = 0.007), and (1 − ODI) (β = −0.06, 95% CI = [−0.10; −0.01], *p* = 0.020) all differed significantly between edematous and contralateral WM, while P_2_ did not (β = 0.01, 95% CI = [−0.09; +0.11], *p* = 0.72). Notably, the magnitudes and directions of change differ across metrics: FA, single-shelled FW-FA, and multi-shelled FW-FA decreased substantially in edema (~44%, 44%, and 36%, respectively), pushing values toward ranges characteristic of GM; alternatively, (1 − ODI) increased only slightly (~8%), with values remaining within ranges characteristic of coherent WM [27]. Group-level results are summarized in Figure 3. Numerical differences were additionally assessed and are shown on a participant-by-participant basis in the Supplementary Material (see Figure S2).

### 3.2. Tractography Outcomes

**Figure 4:**
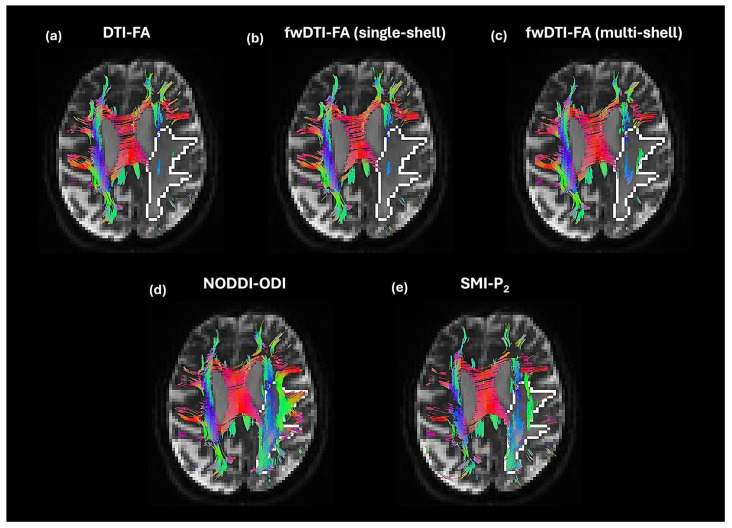
Representative whole-brain WM tractography for a participant viewed along an axial slice using the algorithm FACT with primary direction vectors scaled by standard (**a**) DTI’s FA, (**b**) single-shell FW-DTI’s FA, (**c**) multi-shell FW-DTI’s FA, (**d**) NODDI’s (1 − ODI), and (**e**) the SM’s P_2_. Streamlines were seeded within the boundaries of the brain parenchyma using the brain mask as a seed map. The location of highlighted peritumoral edema is outlined in white. Streamlines are color-coded as follows: red = right–left, blue = superior–inferior, and green = anterior–posterior.

**Figure 5:**
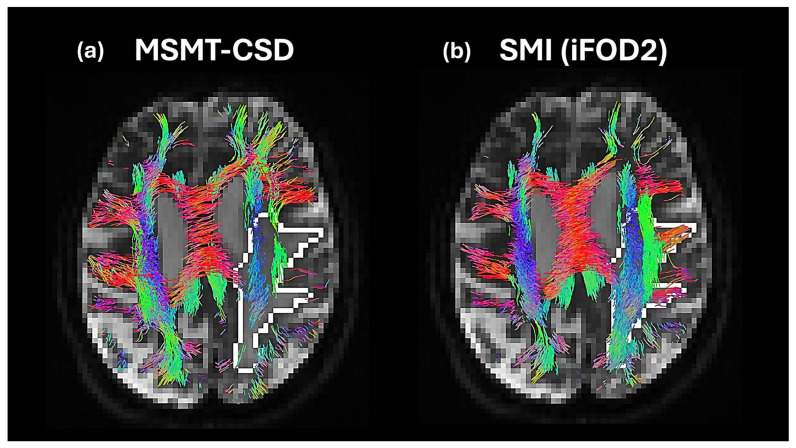
Representative WM tractography viewed along an axial slice using the probabilistic algorithm iFOD2, using (**a**) fODFs calculated from MSMT-CSD and (**b**) fODF outputs in the SH basis from SM fitting. Streamlines are color-coded as follows: red = right–left, blue = superior–inferior, and green = anterior–posterior.

Tractography results showed clear visual differences in fiber tracking continuity through edematous tissue across models. Tractography based on the scaled eigenvectors derived from the DTI and both FW-DTI models failed to generate tracts within edema, resulting in sparse or absent streamlines in the peritumoral region. In contrast, the biophysical compartment-based models NODDI and the SM better generated streamlines through white matter in edematous regions. These models recovered streamlines with anatomically expected orientations and trajectories within the edema, preserving the continuity where DTI- and FW-DTI-based methods failed. Figure 4 shows representative deterministic tractography results, highlighting the contrast between diffusion modeling-based tractography methods within the edematous regions. Similarly, Figure 5 shows representative tractography results using the probabilistic iFOD2 tracking algorithm, where SM fODFs better traced fibers with an anatomically expected structure in edema compared to MSMT-CSD fODFs (representative tractography results for all subjects are visualized in the Supplementary Material, Figures S3 and S4).

### 3.3. Neurite Density and Isotropic Compartment Metric Details

**Figure 6:**
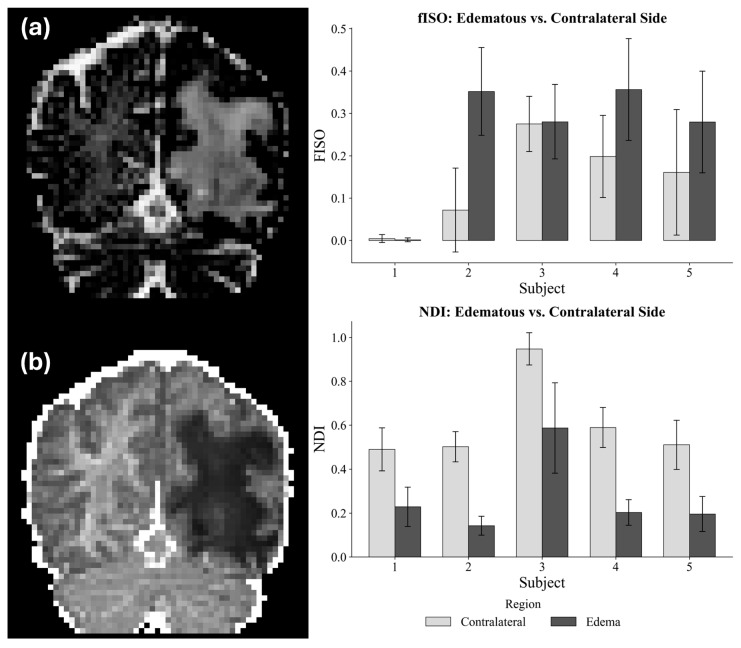
(**a**) Representative NODDI-derived isotropic volume fraction (fISO) and (**b**) neurite density index (NDI) maps for a participant with meningioma-induced edema. Plots represent voxel-wise (**a**) fISO and (**b**) NDI values in regions of edema versus regions in the contralateral healthy-appearing side of the brain. fISO values were found to be higher in the edematous region, likely due to an increased volume of tumor-induced edema. In contrast, NDI values were found to be lower in edematous regions.

**Figure 7:**
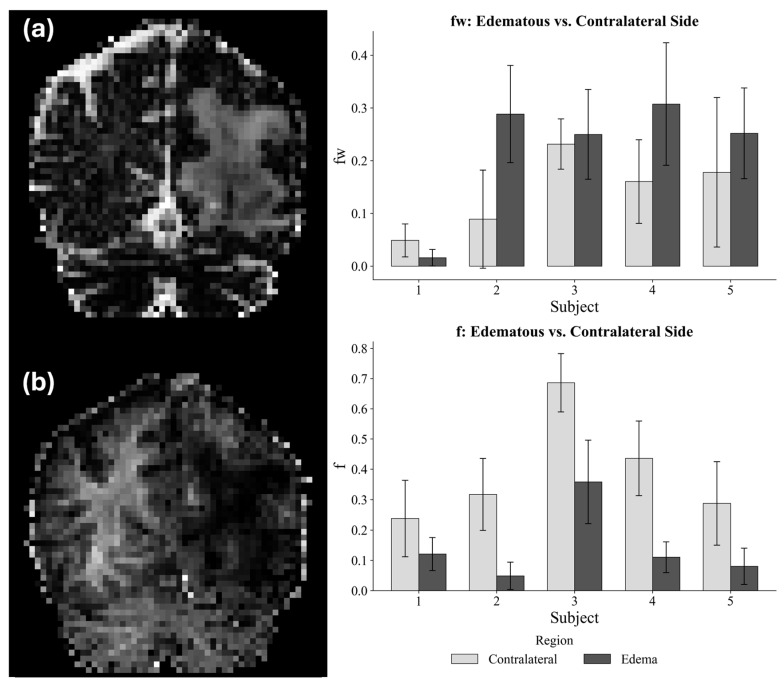
(**a**) Representative SM-derived free-water fraction (fw) and (**b**) axonal water fraction (f) maps for a participant with meningioma-induced edema. Plots represent voxel-wise (**a**) fw and (**b**) f values in regions of edema versus regions in the contralateral healthy-appearing side of the brain. fw values were found to be higher in the edematous region, likely due to an increased volume of tumor-induced edema. In contrast, f values were found to be lower in edematous regions.

To contextualize the behavior of NODDI- and SM-derived compartment fraction metrics in edema, we examined NODDI- and SM-derived metrics using Equation (1), along with a statistical comparison. Figure 6 shows a representative fISO map (6a) and NDI (6b) map from a participant with left-hemisphere edema, along with summary group analysis plots. Edematous regions demonstrated elevated fISO (123.0 ± 152.9% increase relative to contralateral WM, *p* = 0.10) and significantly reduced NDI (58.0 ± 13.0% decrease, *p* = 0.0001). Figure 7 shows the corresponding SM-derived free-water fraction (fw; 7a) and axonal water fraction (f; 7b) maps and summative group analysis plots. Edematous regions exhibited elevated fw (86.6 ± 83.0% increase, *p* = 0.13) and significantly reduced f (65.7 ± 16.4% decrease, *p* = 0.003) relative to contralateral WM.

## 4. Discussion

Here, we focus on using advanced diffusion modeling techniques to identify WM tracts in a controlled study containing only non-infiltrative meningioma cases where no/minimal WM disruption or tumor invasion is expected in regions of edema [27,53]. Consistent with this, radiological evaluation by a board-certified neuroradiologist verified that the regions of edema did not overlap with tumor margins or areas of suspected WM compromise and that there were no radiologic signs of tissue loss attributable to mass effect or swelling. Throughout, we frame our findings as evidence for improved WM tract identification in edema rather than the characterization of WM integrity per se, an important distinction given that microstructural integrity cannot be confirmed without histological validation in this patient population.

Consistent with our hypotheses, more advanced diffusion biophysical modeling techniques (represented here by NODDI and the SM) provided improved WM visualization in edema; this is true in both the scalar map and tractography representations (please refer to Figure 1, Figure 3, Figure 4 and Figure 5 and Table 1). This comes from these methods’ abilities to explicitly separate isotropic and anisotropic diffusion components within a voxel.

DTI-derived FA showed large percent differences between edematous and contralateral WM (~44%, range: 9–68%), indicating a high sensitivity to edema and an inability to identify WM through edema. FW-FA also reflected large percentage differences between edematous and contralateral WM (single-shell: ~44%, range: 7–72%; multi-shell: ~36%, range: 15–57%), which reflect the established limitations of free-water correction with both single- and multi-shelled approaches when the fast diffusion component deviates from the predicted free diffusivity of water (*D* = 3 × 10^−3^ mm^2^/s) [24,26]. In both cases, the reduction is intrinsic to the metric. FA and FW-FA are computed from a tissue tensor whose anisotropy is directly affected by isotropic partial volume contamination, so partition error (or single-tensor averaging in the case of DTI) propagates directly into the reported anisotropy value.

In contrast, NODDI-derived (1 − ODI) and SM-derived P_2_ showed substantially smaller shifts in the case of (1 − ODI) (~8% relative *increase*, *p* = 0.020) and no measurable shift in the case of P_2_ (*p* = 0.72). Both metrics’ robustness to edema arises because they are estimated jointly with the free-water and extra-neurite compartments, better isolating the stick compartment from isotropic contamination. The divergence between (1 − ODI) and P_2_, however, suggests a NODDI-specific model behavior that biases (1 − ODI) towards a paradoxical *increase* in fiber coherence. This, of course, is unlikely to be a physiologic phenomenon. NODDI couples the extra-neurite tensor’s anisotropy to orientation dispersion via the tortuosity model [27], so when the actual diffusivity in edematous fluid deviates from the assumed value (*D* = 3 × 10^−3^ mm^2^/s), signal mis-partitioning between isotropic and extra-neurite compartments biases the ODI toward lower estimates (driving (1 − ODI) and apparent fiber coherence higher). Similar effects appear in the ODI maps of Chong et al. [44] in peritumoral regions near the corticospinal tract. The SM does not impose this directional coupling and allows the intra-axonal and extra-axonal diffusivities (but not the free-water diffusivity) to be fit freely, allowing P_2_ to remain stable across regions. Both (1 − ODI) and P_2_ values nonetheless remained close to within-subject contralateral measurements (Table 1) and to typical values reported in healthy WM [27,28].

NODDI and SM volume fraction outputs further contextualize these findings. Edematous regions exhibited elevated isotropic and free water fractions (fISO, fw) and reduced intra-neurite/intra-axonal fractions (NDI, f) (Figure 6 and Figure 7). It is important to note that these are fractional, not absolute, volumes. NDI is defined as an intra-neurite signal divided by the sum of intra-neurite and extra-neurite signals (excluding the isotropic compartment), whereas f is defined as an intra-axonal signal divided by the sum of all compartmental signals. Vasogenic edema increases the extracellular water volume, which inflates both the free-water and extra-neurite compartments. This has the effect of increasing the denominators of the NDI and f calculations and thus reducing the fractional values without requiring a reduction in absolute intra-neurite content. The observed reductions in NDI and f are attributed here to an edema-induced increase in extracellular fluid rather than neurite loss. The shared fixed free-water diffusivity assumption discussed above is an additional contributor that can drive mis-partitioning of the signal across compartments when actual diffusivities deviate from the model-assumed values, with volume fraction outputs most directly affected because they are computed from compartmental sizes.

The fiber orientation reconstructions derived from these biophysical models carry the same advantage as the scalar maps. Improved tractography for NODDI- and SM-based tract estimates can be observed in Figure 4, Figures S3 and S4. We show that orientation scalar mapping from NODDI and the SM (ODI and P_2_) can be paired with this directional information to more reliably trace edematous WM tracts. These improvements may reflect more accurate directional fittings atop scalar metrics more sensitive to WM in edema, further underscoring the advantages of these biophysical approaches.

Previous work [44,54,55,56,57,58,59,60,61,62] has compared diffusion metrics in and around brain tumors, finding reduced FA in edematous and tumoral regions and highlighting FA’s limited reliability in WM identification near lesions. Notably, previous work by Chong et al. [44] highlighted the use of NODDI to enhance tractography in regions affected by peritumoral edema, demonstrating that NODDI-based tractography could visualize fiber tracts not detectable by standard DTI. However, interpretation of these studies is limited by heterogeneous cohorts that combine infiltrative (glioma) and non-infiltrative (meningioma) tumors. This mixing can conflate edema-related partial volume effects with true tract disruption. While both tumor types generate edema, glioma additionally infiltrates and degrades WM tract integrity and further alters diffusion profiles beyond the effects of edema alone [21,22]. To build on this work, we restricted our analysis to meningioma [29,30,31,63] to eliminate cellular infiltration as a confounder and isolate the impact of edema only on diffusion signals.

A concern with any tractography analysis is the generation of false positives via stopping thresholds that are too liberal. We addressed this concern in two ways. First, and to avoid circular threshold tuning that could bias results, tractography thresholds were calibrated on an independent cohort of five independent healthy older adult controls separate from the meningioma participants. We implemented a Dice optimization approach that produced thresholds in good agreement with those established in the literature [64,65]. Second, all tractography reconstructions were independently reviewed by a board-certified neuroradiologist, who confirmed the anatomic plausibility of the streamline trajectories. These steps reduce, but do not eliminate, the possibility of false positive streamlines. Notably, the bias in (1 − ODI) may still present a small degree of false positive generation despite the above efforts to mitigate false positive streamlines. We refrained from extending beyond our *a priori* methodologic boundaries described above to mitigate bias in our interpretation; however, the optimization of (1 − ODI) stopping thresholds will certainly be the topic of future study. In total, the tractography results should be interpreted as qualitative proof-of-concept evidence of improved streamline continuity through edema rather than validated reconstructions of true axonal trajectories.

While P_2_ in edematous and non-edematous WM showed no measurable differences, it should be noted that this study does not assert statistical equivalence. The low statistical power of this proof-of-concept cohort (*n* = 5) is a limitation of this study, and a larger cohort would be required to formally establish equivalence. We note, however, that the central claim of this work does not require statistical equivalence. The finding is that NODDI and the SM are useful for identifying WM tracts in edema, where DTI and FW-DTI are less so. This claim extends across both scalar map and tractography results. Visual assessment and atlas overlays aided the interpretation of both scalar and tractography maps, but these approaches remain surrogate validations, as WM biopsies are not acquired in human meningioma cases, and thus, histological confirmation of healthy WM is not accessible. We additionally note that no radiologic evidence of Wallerian degeneration or encephalomalacia was observed in any of the five participants by an independent board-certified neuroradiologist, reducing (but not eliminating) the possibility that observed diffusion changes reflect secondary structural degradation rather than edema alone.

Extending beyond deterministic FACT-based tractography, we evaluated two probabilistic tractography approaches. Probabilistic multi-shell, multi-tissue constrained spherical deconvolution (MSMT-CSD) [50] tractography also failed to propagate streamlines through edematous regions in a manner comparable to DTI- and FW-DTI-based tracking. However, probabilistic tractography driven by SM-derived fODFs successfully propagated streamlines through edematous regions where MSMT-CSD-based tractography did not (Figure 5). MSMT-CSD deconvolves the multi-shell signal against fixed-shape response functions for WM, GM, and CSF calibrated from normal-appearing voxels in the dataset, and these response functions no longer match the underlying tissue in edema. Alternatively, and as described above, the SM jointly estimates compartmental parameters with the fODF on a voxel-wise basis, partitioning out the isotropic contribution during fitting, so the resulting fODF reflects the anisotropic signal components with minimal isotropic contamination. Tractography results across deterministic and probabilistic approaches are shown for all participants across two representative views in the Supplementary Material (Figures S3 and S4).

Our DTI protocol used standard clinical single-shell data (b = 0 and 1000 s/mm^2^, 18 directions) extracted from our larger multi-shell dataset, ensuring that this analysis compares to common practice and enabling comparison with NODDI and the SM within typical clinical constraints [66,67,68]. For the implementation of DTI, we used 18 directions, because that is what was acquired in the b = 1000 shell from our multi-shell diffusion sequence. For comparison, we show that results from this 18-direction, single-shell dataset are comparable to a clinical standard 30-direction, single-shell acquisition (Supplementary Material, Figure S1). Similarly, we investigated two implementations of FW-DTI, including a single-shelled variant using spatial regularization well-explored in the literature [69,70,71,72], as well as a multi-shelled extension of FW-DTI previously shown to be more well-posed than its single-shelled counterpart [73].

Historic challenges to deploying these methods include the collection of multi-shell datasets, which traditionally require increased acquisition times. However, the emergence of multiband [74], parallel imaging [75], and compressed sensing [76] acquisition schemes has dramatically reduced acquisition times to allow dMRI datasets satisfying more advanced diffusion modeling techniques to be acquired in approximately five minutes [54]. Future studies will focus on the more complex cases of invasive tumors like high-grade gliomas. Complementary rodent studies focusing on invasive brain tumors are ongoing in this laboratory and seek to validate dMRI results against histologic and assay studies. Finally, future work will also involve: (i) expansion into other biophysical models of diffusion in brain tissue with great data demands, including variations on the Standard Model [28] like the Soma And Neurite Density Imaging (SANDI) [77] model and the Neurite Exchange Imaging (NEXI) [78], and (ii) deeper investigation into probabilistic tractography with compatible outputs like those from the SM.

## 5. Conclusions

In this proof-of-concept study, we compared the performance of DTI, FW-DTI, NODDI, and the SM for detecting WM tracts in regions of peritumoral edema. A controlled experiment using only non-infiltrative meningioma cases was used to mitigate tract degradation and tumor cell infiltration associated with invasive glioma cases. NODDI’s (1 − ODI) and the SM’s P_2_ values in edematous regions remained close to within-subject contralateral measurements and typical values reported in healthy WM [27,28], contrasting with the substantial reductions in FA and FW-FA. Similarly, NODDI and the SM successfully improved fiber tracking continuity through edema, whereas DTI- and FW-DTI-based methods did not. Beyond deterministic tractography, probabilistic tractography driven by SM-derived fODFs successfully propagated streamlines through edematous regions where multi-shell, multi-tissue CSD-based tractography failed, thereby showing that the central observation also appears in a probabilistic SM-derived fODF implementation. These findings indicate that biophysical, multi-compartment models are more robust to isotropic partial volume effects than tensor-based approaches and provide a more reliable framework for identifying WM tracts in regions of edema, with implications for preoperative neurosurgical planning where conventional methods may fail.

## Figures and Tables

**Table 1:** Mean ± SD of standard fractional anisotropy (FA), single-shelled (SS) and multi-shelled (MS) free-water-corrected fractional anisotropy (FW-FA), inverted orientation dispersion index (1 − ODI), and the second-order rotational invariant of the fODF (P_2_) for white matter in edematous (Ipsi) vs. contralateral healthy-appearing (Contra) regions with percentage differences (%Diff).

Patient		1	2	3	4	5
FA	Ipsi	0.32 ± 0.08	0.14 ± 0.04	0.31 ± 0.09	0.16 ± 0.07	0.14 ± 0.04
	Contra	0.46 ± 0.13	0.44 ± 0.11	0.34 ± 0.12	0.34 ± 0.14	0.33 ± 0.15
	%Diff	−30.43%	−68.18%	−8.82%	−52.94%	−57.58%
FW-FA (SS)	Ipsi	0.55 ± 0.12	0.20 ± 0.07	0.52 ± 0.13	0.24 ± 0.11	0.21 ± 0.07
	Contra	0.74 ± 0.15	0.71 ± 0.17	0.56 ± 0.18	0.55 ± 0.22	0.52 ± 0.21
	%Diff	−25.68%	−71.83%	−7.14%	−56.36%	−59.62%
FW-FA (MS)	Ipsi	0.40 ± 0.08	0.23 ± 0.06	0.52 ± 0.11	0.31 ± 0.12	0.24 ± 0.08
	Contra	0.53 ± 0.12	0.53 ± 0.14	0.61 ± 0.17	0.52 ± 0.18	0.43 ± 0.18
	%Diff	−24.53%	−56.60%	−14.75%	−40.38%	−44.19%
1 − ODI	Ipsi	0.86 ± 0.04	0.77 ± 0.07	0.73 ± 0.07	0.75 ± 0.14	0.72 ± 0.14
	Contra	0.80 ± 0.07	0.77 ± 0.09	0.64 ± 0.12	0.68 ± 0.14	0.66 ± 0.13
	%Diff	7.50%	0.00%	14.06%	10.29%	9.09%
P_2_	Ipsi	0.53 ± 0.08	0.33 ± 0.05	0.34 ± 0.07	0.36 ± 0.12	0.30 ± 0.10
	Contra	0.49 ± 0.09	0.46 ± 0.09	0.27 ± 0.10	0.35 ± 0.12	0.36 ± 0.11
	%Diff	8.16%	−28.26%	25.93%	2.86%	−16.67%

## Data Availability

Raw imaging data are not publicly available to protect participant privacy and satisfy approved institutional review board protocols. Anonymized, derived quantitative features extracted from the imaging data are available from the corresponding author upon reasonable request.

## Supplementary Materials

The following supporting information can be downloaded at: https://www.mdpi.com/article/10.3390/tomography12060078/s1, Figure S1: Comparison of 18-direction and 30-direction single-shell DTI outputs; Figure S2: Scalar metric comparisons by participant and approach; Figure S3: Deterministic and probabilistic tractography results for all participants; Figure S4: Deterministic and probabilistic tractography results for all participants, alternate view.
