# Supplementary material for: Biophysical Diffusion MRI Models Better Identify White Matter Tracts in Edema
Source: Tomography. 2026 May 25;12(6):78. doi: 10.3390/tomography12060078 (PMC13306668; doi:10.3390/tomography12060078)
Supplement: Supplementary file 1 [file tomography-12-00078-s001.zip › tomography-4182327-supplementary.pdf]

Supplementary Material

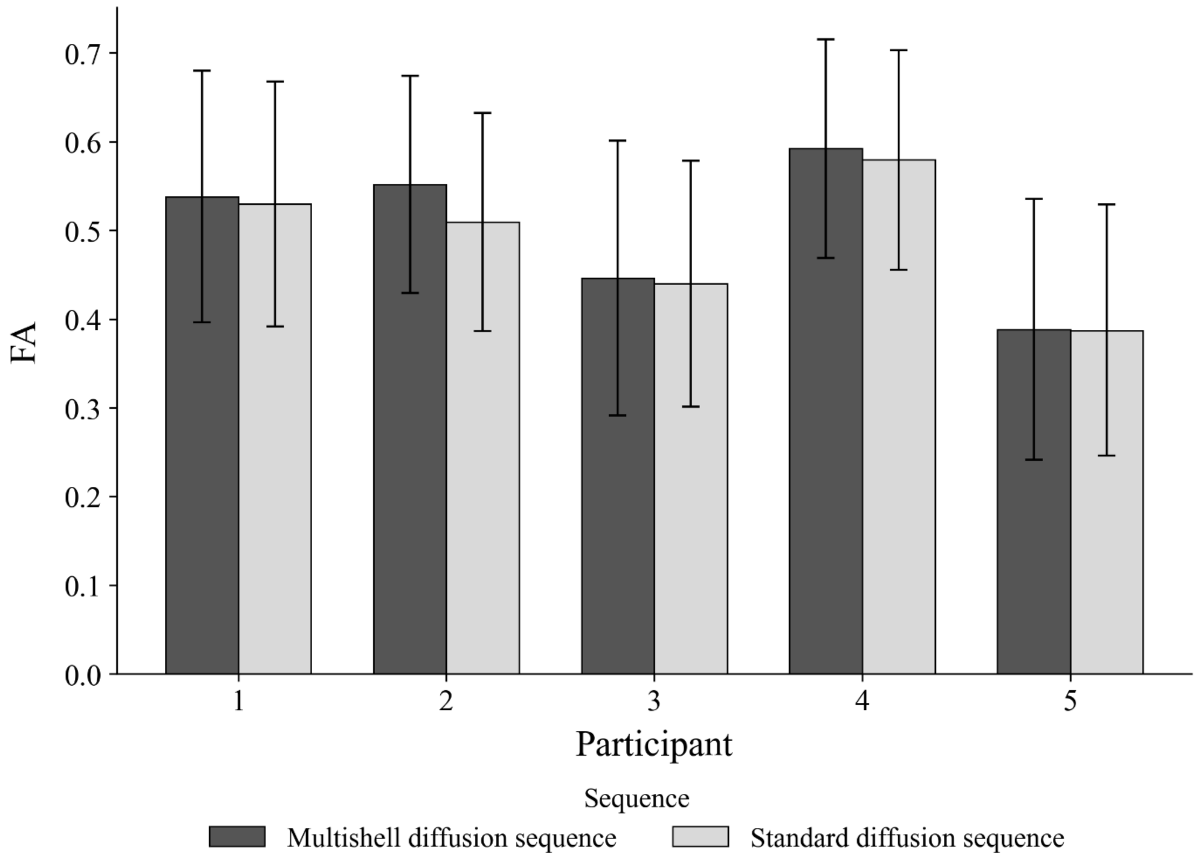

Figure S1. Bar plot comparing fractional anisotropy (FA) values derived from the standard diffusion sequence and multi-shell diffusion sequence across participants in the corpus callosum.

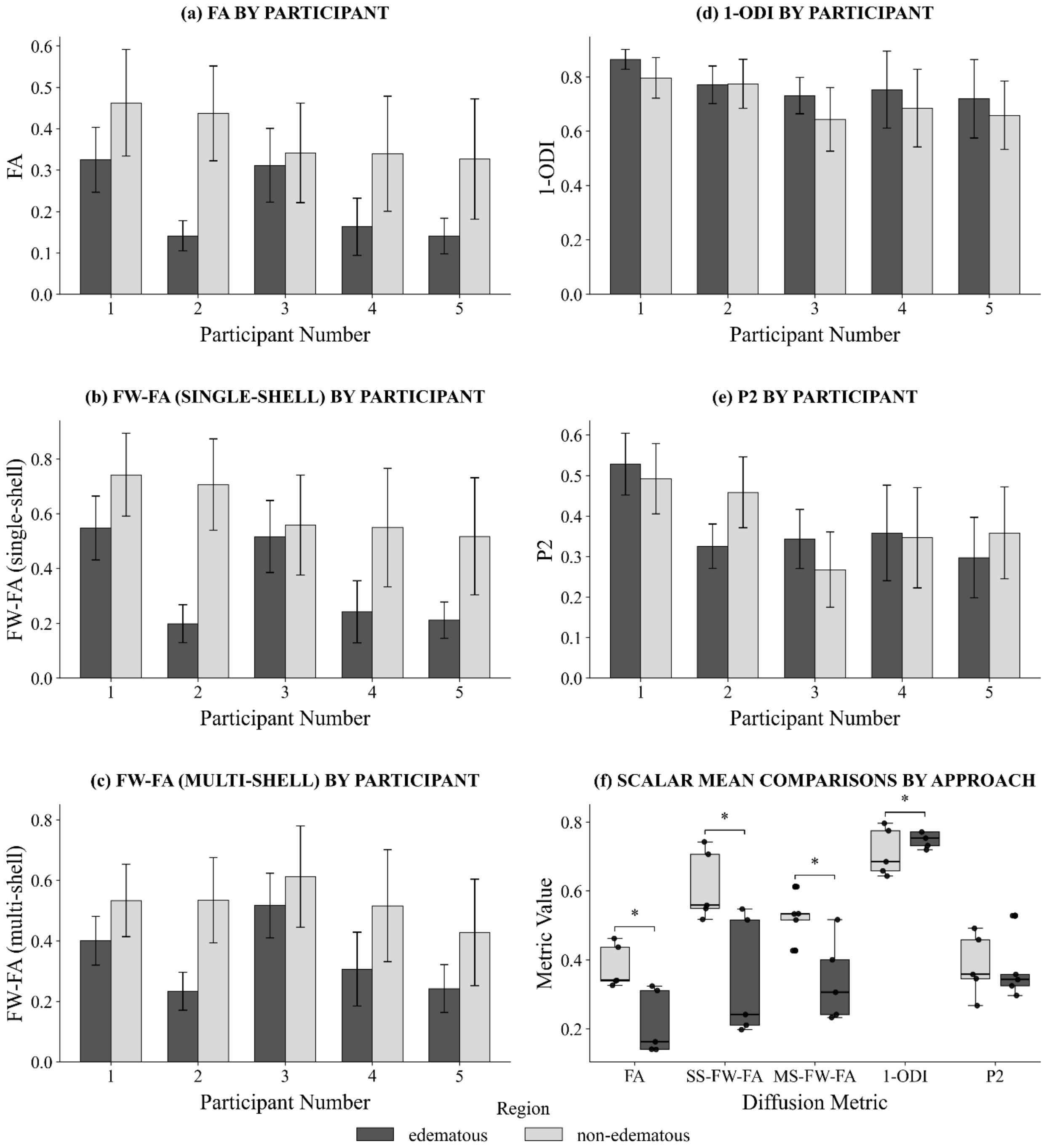

Figure S2. Metrics in white matter between edematous and non-edematous regions derived from (a) standard FA maps, (b) single-shelled FW-FA, (c) multi-shelled FW-FA, (d) 1-ODI maps, (e) P_2 maps by participant, and (f) statistical comparisons between aggregated edematous and non-edematous regions by diffusion metric.

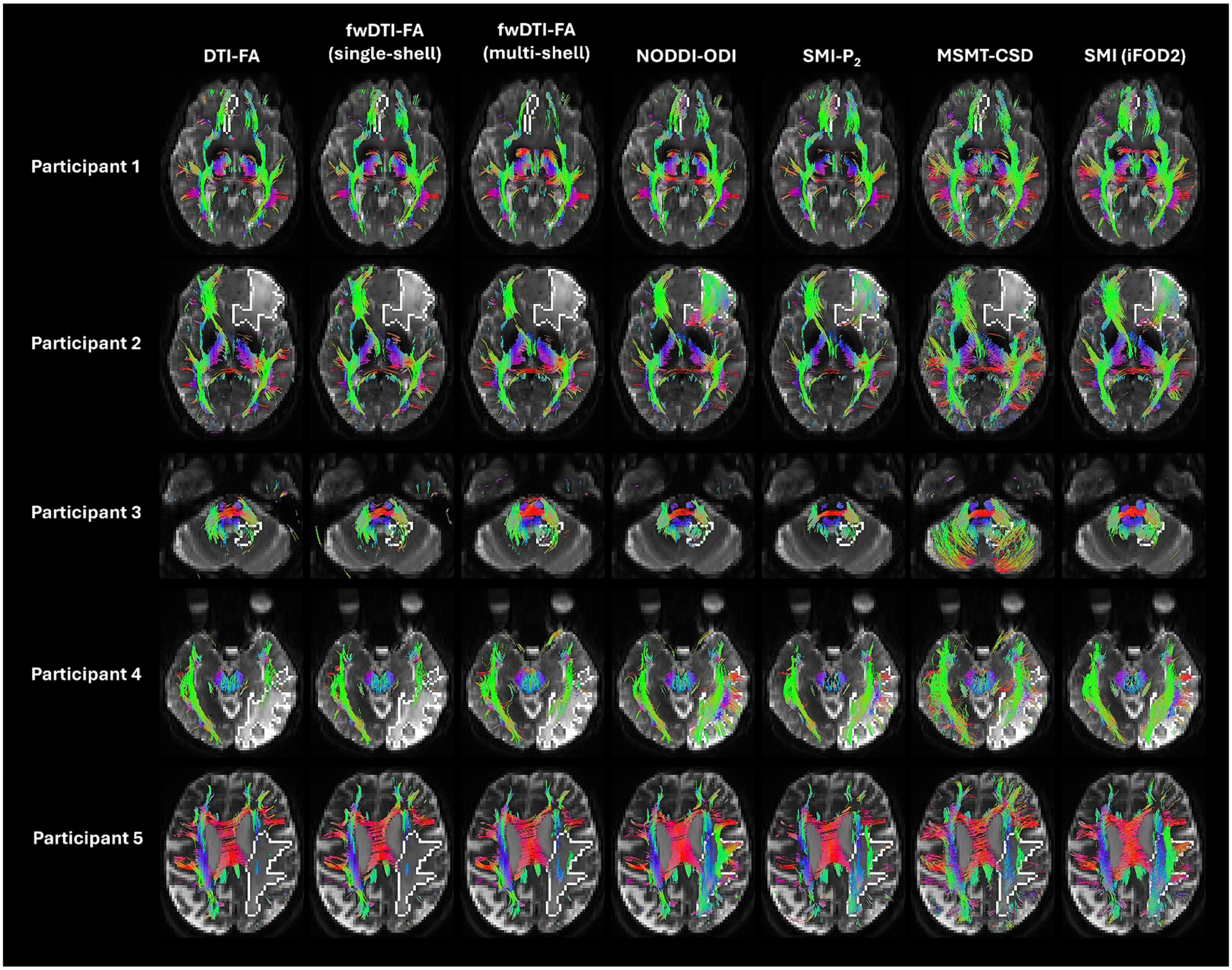

Figure S3. Comparison of whole brain WM tractography for all participants viewed using the algorithm FACT with primary direction fields scaled by standard DTI's FA, single shell FW-DTI's FA, multi-shell FW-DTI's FA, NODDI's ODI, and SM's P_2, alongside probabilistic algorithm iFOD2 driven by fODFs from MSMT-CSD and SM.

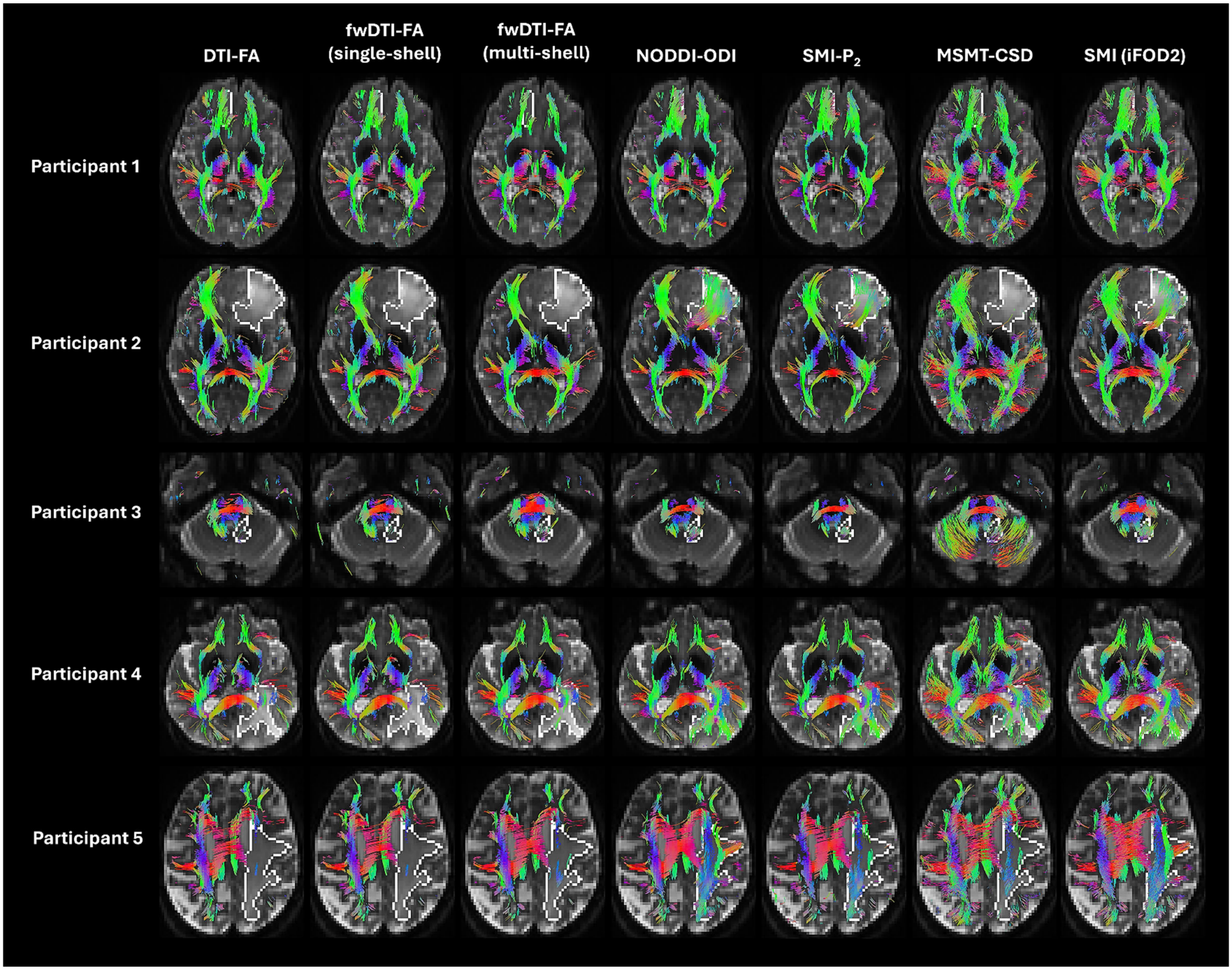

Figure S4. Comparison of whole brain WM tractography for all participants viewed from an alternative axial slice. Shown are deterministic FACT tractography results from scaled primary direction inputs from DTI, single- and multi-shelled FW-DTI, NODDI, and SM, along with probabilistic iFOD2 tractography results from fODFs calculated from MSMT-CSD and SM.
